# Pre-frail older adults show improved cognition with *StayFitLonger* computerized home–based training: a randomized controlled trial

**DOI:** 10.1007/s11357-022-00674-5

**Published:** 2022-10-21

**Authors:** Sylvie Belleville, M. Cuesta, M. Bieler-Aeschlimann, K. Giacomino, A. Widmer, A. G. Mittaz Hager, D. Perez-Marcos, S. Cardin, B. Boller, N. Bier, M. Aubertin-Leheudre, L. Bherer, N. Berryman, S. Agrigoroaei, J. F. Demonet

**Affiliations:** 1 Research Centre, Institut Universitaire de Gériatrie de Montréal, CIUSSS du Centre-Sud-de-L’Île-de-Montréal, 4565, Queen-Mary Road, Montreal, Quebec H3W 1W5 Canada; 2 Université de Montréal, Montreal, Canada; 3 Leenaards Memory Centre and Infections Disease Service, University Hospital of Lausanne, Lausanne, Switzerland; 4 MindMaze, SA, Lausanne, Switzerland; 5 HES-SO Valais-Wallis, School of Health Sciences, Loèche-les-Bains, Switzerland; 6 HES-SO Valais-Wallis, School of Management, Sierre, Switzerland; 7 Université du Québec à Trois-Rivières, Trois-Rivieres, Canada; 8 Université du Québec à Montréal, Montreal, Canada; 9 Montréal Heart Institute, Montreal, Canada; 10 Psychological Sciences Research Institute, Université catholique de Louvain, Louvain-la-Neuve, Belgium

**Keywords:** Cognitive training, Physical training, Home-based computerized training, Frailty, Cognitive prevention

## Abstract

Multidomain interventions have shown tremendous potential for improving cognition in older adults. It is unclear if multidomain interventions can be delivered remotely and whether remote intervention is beneficial for older adults who are vulnerable or at risk of cognitive decline. In a 26-week multi-site, home-based, double-blind, randomized controlled trial, 120 cognitively healthy older adults (75 robust, 45 pre-frail; age range = 60–94) recruited from Switzerland, Canada, and Belgium were randomized to receive either the StayFitLonger (SFL) computerized multidomain training program or an active control intervention. Delivered on tablets, the SFL intervention combined adapted physical exercises (strength, balance, and mobility), cognitive training (divided attention, problem solving, and memory), opportunities for social and contributive interactions, and psychoeducation. The active control intervention provided basic mobilization exercises and access to video games. Cognitive outcomes were global cognition (*Z*-scores of attention, verbal fluency, and episodic memory for nondemented older adults; ZAVEN), memory, executive function, and processing speed. Linear mixed model analyses indicated improved performance on the ZAVEN global cognition score in the SFL group but not in the active control group. Stratified analyses by frailty status revealed improved ZAVEN global cognition and processing speed scores following SFL in the pre-frail group but not in the robust group. Overall, the study indicates that a computerized program providing a multidomain intervention at home can improve cognition in older adults. Importantly, pre-frail individuals, who are at higher risk of cognitive decline, seem to benefit more from the intervention. Trial registration: ClinicalTrials.gov, NCT037519 Registered on January 22, 2020—Retrospectively registered, https://clinicaltrials.gov/ct2/show/NCT04237519.

**Supplementary Information:**

The online version contains supplementary material available at 10.1007/s11357-022-00674-5.

## Introduction

Age-related cognitive decline is associated with modifiable risk factors that can be addressed with non-pharmacological approaches [1]. For this reason, multidomain prevention programs that target a subset of modifiable factors have been developed to promote cognitive health in older adults [2]. The positive impact of multidomain interventions has been observed in a few studies that evaluated their effect in older adults at risk of cognitive decline. For example, the FINGER study, which combined face-to-face physical activity with computerized cognitive training [3], reported a positive effect on overall cognition and a reduced risk of cognitive decline. Thus, prevention programs have enormous potential to protect older adults from the deleterious effects of brain aging on cognition, which can ultimately preserve their independence [2, 4].

While prior studies reported encouraging effects, some issues remain to be addressed. The first relates to the accessibility and flexibility of face-to-face multidomain interventions. Older adults may have mobility challenges or live in remote areas without access to community resources providing face-to-face interventions. With the increase in technological literacy among older adults, there has been considerable recent interest in developing computerized programs to deliver home-based interventions. These interventions can increase flexibility of use, reduce costs, and thus facilitate the scaling up of interventions. Computerized programs allow for real-time feedback on performance, control of item timing, and gamification, among other advantages. Surprisingly, only a few studies have evaluated at-home physical activity training or multidomain programs [5–9].

A second important issue to address is interindividual variability in response to computerized multidomain interventions. From a personalized medicine perspective, it is important to know the responders and their characteristics. In the present study, we examined efficacy as a function of frailty status–defined as a state of heightened vulnerability due to impairment of multiple systems [10, 11]. Frailty is an important predictor of loss of independence and cognitive decline and thus, a highly relevant marker of vulnerability in old age [12]. Predictions is based on two frameworks: The compensation/reserve model posits that vulnerable older adults will benefit the most from these interventions, while the magnification model posits that cognitive improvement following an intervention involves brain plasticity and that the fittest individuals will benefit most because their brain is more plastic [13, 14].

Here, we report on a 26-week double-blind parallel-group randomized controlled trial (RCT), which examined the cognitive effects of the home-based computerized multidomain intervention StayFitLonger (SFL), combining physical exercise and cognitive training, compared to an active control condition. Results are reported for the full sample and then separately for pre-frail and robust older adults. We hypothesized that the SFL group would have a larger pre-post intervention effect than the control group. As the compensation model has been most often supported, we predicted a larger SFL advantage in pre-frail participants compared to robust ones.

## Methods

The study was pre-registered (ClinicalTrials.gov Identifier: NCT04237519) and follows the recommendations of the updated Consolidated Standards of Reporting Statement [15, 16]. All procedures were reviewed and approved by the Research Ethics Board (REB) in each country: Switzerland: REB Canton de Vaud (application #2018–01898, last approval December 4 2018); Canada: REB vieillissement-neuroimagerie of the CIUSSS-CSMTL (application #18–19–29, last approval December 14 2018); Belgium: REB Cliniques Universitaires Saint-Luc, UCLouvain, Bruxelles (application #B403201941535, last approval October 15 2019). The nature, benefits, and risks of the study were explained to all subjects, and their written informed consent was obtained prior to participation. The cognitive outcomes reported here were identified as secondary outcomes. The primary outcome and secondary psychosocial outcomes are reported separately. As the protocol of the SFL study was published previously [17], only the main aspects of the methods are described.

### Design

The efficacy trial was a 26-week double-blind parallel group multi-centric RCT. Participants were randomized to either the SFL home-based computerized multidomain intervention or a home-based active control intervention. Outcome measures were collected at pre-training (PRE; within 6 weeks prior to the start of the intervention) and post-training (POST, within 4 weeks following the end of the intervention). Randomization was done independently from the research team with a 1:1 ratio stratified based on the frailty status using REDCap. Participants were blinded to the nature of their intervention (experimental vs comparator), and assessors were blinded to the hypotheses and the participants’ assignment. Statistical analyses were blinded to the intervention condition.

### Study population and entry criteria

**Fig. 1:**
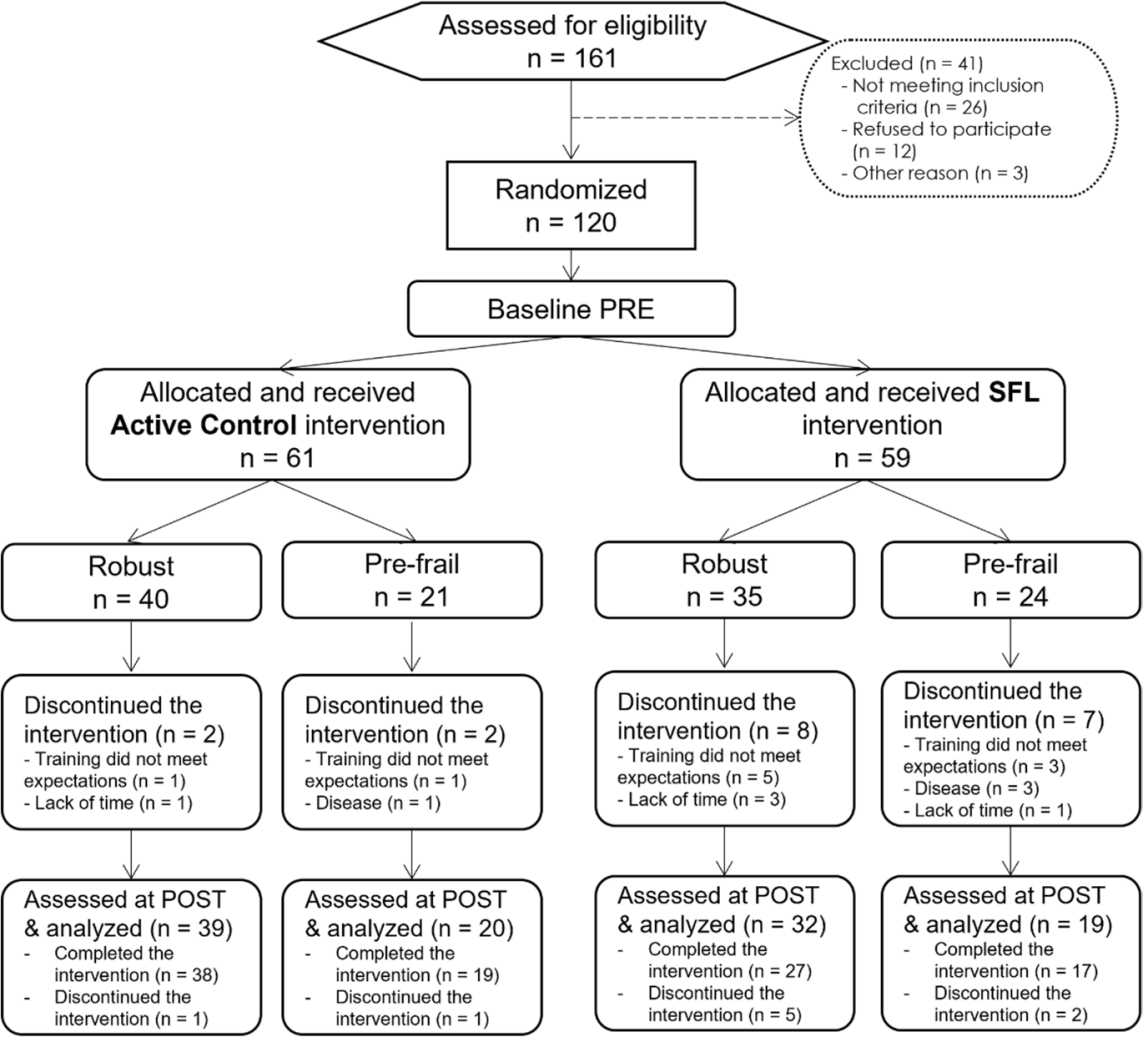
Flow diagram of participants’ progress in the efficacy study

Participants were recruited from three sites: Centre Leenaards de la mémoire – Centre hospitalier universitaire Vaudois (CHUV), Switzerland; Institut universitaire de gériatrie de Montréal of the Centre intégré universitaire de santé et de services sociaux Centre-Sud-de-l’Île-de-Montréal (CIUSSS-CSMTL), Canada; and Brusano and Centre Public d’Action Sociale (CPAS) of Woluwe-Saint-Lambert, Belgium. The participant flow is shown in Fig. 1. Of the 161 participants tested for eligibility, 120 were randomized (64 in Switzerland, 32 in Canada, and 24 in Belgium). Fifty-nine were allocated to the SFL intervention and 61 to the active control. As participants from the Belgian site were included during the COVID-19 pandemic, the introductory courses, which were provided in group sessions in the other sites [17], were provided to participants through videos followed by a home visit from the instructor.

Included participants were fluent French-speaking community-dwelling adults aged 60 years and over with normal scores on the 4-Instrumental Activities of Daily Living (4-IADL) scale [18], a score ≥ 26 on the Montreal Cognitive Assessment (MoCA) [19], a score < 3 on the Fried’s frailty index [11], no motor or vision problems, no current neurological or psychiatric diagnoses (e.g., Parkinson’s disease), and access to a wireless Internet connection at home. Participants were identified as either robust (score of 0) or pre-frail (score of 1 or 2) based on Fried’s index.

### Interventions

Interventions were provided on a tablet (Samsung Galaxy Tab S2) and took place at home. Participants received occasional home visits and monthly phone calls to monitor their use and address any problems with the program. The mean overall time (in hours) that each group spent using the program was recorded and will be only briefly summarized here as it will be the topic of a separate publication on adherence (see design paper [17]).

#### SFL intervention

The SFL intervention included physical and cognitive training activities. The physical exercises (*Exercise*) focused on strength, balance, and mobility with various difficulty levels [20]. Cognitive training included activities for divided attention [21], problem solving [22, 23], and memory [24]. To increase adherence and social interactions, participants had access to a moderated *Chat Room*, the possibility to create material for the activities, psycho-educational content, and gamification elements (e.g., rewards, leaderboards). A customizable virtual guide provided participants with instructions, reminders, and feedback. Participants were asked to engage in physical exercise at least 3 days per week for 30–45 min and cognitive exercise for at least three 15-min sessions per week.

#### Active control intervention

The active control intervention had similar structure, timing, and organization as the SFL program. Physical exercises included advice and tips to stay physically active and exercises to train strength, mobility, and balance of the upper and lower extremities. Unlike the SFL, the active control only had a limited number of physical exercises and did not include interactive videos, personalization, chat rooms, psycho-educational content, or virtual guide. The cognitive activities were commercially available games that did not target specific cognitive processes or strategies [25–29] (e.g., crossword puzzles, Sudoku, maze arcade).

### Outcome variables

Global cognition was measured with an adapted version of the ZAVEN composite score [30], which is the averaged *z*-scores from the delayed free recall of the California Verbal Learning Test (CVLT), delayed recall of the Wechsler Memory Scale-IV logical memory subtest [31], number of correct symbols reported in the Wechsler Adult Intelligence Scale (WAIS)-IV, digit symbol substitution test (DSST) [32], and letter fluency (the letter P at the pre-training and R at the post-training) [33]. An executive function composite score was computed by combining *z*-scores from the letter fluency test, Trail Making Test (TMT) part B-A (time) [34], interference index of the Victoria Stroop Test [35], and number of omissions on the divided attention subtest of the Test of Attention Performance [36]. A memory composite score was obtained from the delayed free recall score of the CVLT [37, 38] and logical memory task. A processing speed composite score was obtained from the TMT part A (time), number of correct answers on the DSST, and the naming condition of the Victoria Stroop Test (time) [39]. Scores were inverted, when necessary, so that larger scores always reflected better performance. The composite scores were computed by standardizing performance on individual tests using the baseline mean and standard deviation (SD) of the entire group. A preliminary internal consistency analysis was conducted to contextualise the measures. This analysis was particularly relevant for the executive function, memory, and processing speed composite scores because they were meant to reflect a single cognitive construct. In contrast, the ZAVEN composite score was developed to diagnose preclinical Alzheimer’s disease and intended to cover multiple cognitive domains to provide greater sensitivity to cognitive decline. Because differences in expectations might explain some of the intervention effects, participant’s expectations were measured at PRE and POST on a 15-item ad hoc questionnaire on a 7-point Likert scale.

### Statistical analyses

The sample size was determined with a Marker Stratified Design^1^ considering a dropout rate of about 25% based on prior studies. All statistical tests were two-tailed with a *p* value < 0.05. Groups were compared for demographics and baseline characteristics with *t*-tests or chi-square analyses. A linear mixed model was used to analyze the intervention effect controlled for age, sex, education, score on MoCA at baseline, and site. The fixed effects were intervention (SFL vs. active control), time (PRE, POST), and their interaction. In the presence of a significant interaction, post hoc comparisons were computed between PRE and POST in each group and mean and confidence intervals were assessed on pre-post change scores. Separate analyses were computed for each outcome. Significant interactions and group differences in favor of the SFL at POST and on change scores were expected if the SFL intervention was more beneficial than the active control. All analyses were first performed with the total sample, followed by separate analyses for pre-frail and robust individuals. A comparison of the clinical and socio-demographic characteristics of participants, who completed vs withdrew from the study, are presented in Supplementary Table 1. To comply with an intention-to-treat (ITT) approach, all randomized participants were included in the model, and the characteristics of participants who withdrew were compared to those remaining in the study (Supplementary Table 1). The effect of sex and other controlled variables on the cognitive outcomes are shown in Supplementary Table 3.

## Results

The mean age of the total sample was 71.33 years (range = 60–94; SD = 5.87). The average score on the MoCA was 28.97 (range = 26–30; SD = 1.17). 79/120 of participants were women. Table 1 reports the baseline characteristics of the sample as a function of the intervention condition and frailty status. There were no differences at baseline for sociodemographic or clinical variables between participants in the SFL vs. active control intervention (Supplementary Table 1). Mean time weekly spent using the program was 2.6 (SD = 0.3) and 3.8 (SD = 0.4) hours for the total SFL group and the active control condition, respectively; 2.4 (SD = 0.4) and 3.4 (SD = 0.7) hours for the pre-frail SFL group and the active control condition, respectively; and 2.7 (SD = 0.4) and 4.0 (SD = 0.4) hours for the robust SFL group and the active control condition, respectively. Cronbach alpha values for the composite scores were 0.54, 0.73, 0.54, and 0.66 for the ZAVEN, memory, executive, and speed composite scores, respectively.

**Table 1:** Participants’ demographic and clinical characteristics at baseline

Group	Characteristics	SFL		Active control		SFL vs Control
		Mean (SD) or N	Range	Mean (SD) or N	Range	*p* value
Total sample (SFL = 59 Control = 61)	Age (y)	70.6 (5.8)	61–82	72.0 (6.7)	60–94	.21
	MoCA score (/30)	29.1 (1.0)	25–30	28.8 (1.3)	26–30	.16
	Sex (male, female)	17, 42	N/A	24, 37	N/A	.22
	Education (Low, Medium, High)	6, 21, 32	N/A	6, 19, 36	N/A	.86
	Site (SW, CA, BE)	33, 15, 11	N/A	31, 17, 13	N/A	.85
	Frailty score (0, 1, 2)	35, 21, 3	N/A	40, 17, 4	N/A	.65
	4-IADL (0, 1, 2, 3, 4)	0, 0, 0, 0, 59	N/A	0, 0, 0, 0, 61	N/A	N/A
	TUG (s)	8.71 (1.23)	5.35–11.15	8.69 (1.95)	5.40–16.50	.95
	HADS – Anxiety (/21)	2.7 (2.3)	0–11	2.3 (2.6)	0–14	.35
	HADS – Depression (/21)	2.9 (2.5)	0–9	2.7 (2.3)	0–14	.57
Pre-frail (SFL = 24 Control = 21)	Age (y)	71.2 (5.5)	61–82	74.8 (8.2)	61–94	.09
	MoCA score (/30)	29.1 (1.0)	27–30	28.8 (1.2)	26–30	.39
	Sex (male, female)	8, 16	N/A	7, 14	N/A	1.00
	Education (Low, Medium, High)	2, 8, 14	N/A	2, 5, 14	N/A	.78
	Site (SW, CA, BE)	14, 7, 3	N/A	11, 6, 4	N/A	.83
	Frailty score (0, 1, 2)	0, 21, 3	N/A	0, 17, 4	N/A	.54
	4-IADL (0, 1, 2, 3, 4)	0, 0, 0, 0, 24	N/A	0, 0, 0, 0, 21	N/A	N/A
	TUG (s)	8.81 (1.18)	6.80–16.50	9.91 (2.36)	6.80–16.50	.05
	HADS – Anxiety (/21)	3.2 (2.8)	0–11	3.1 (3.6)	0.14	.91
	HADS – Depression (/21)	4.2 (3.0)	0–9	3.2 (3.0)	0–14	.30
Robust (SFL = 35 Control = 40)	Age (y)	70.2 (4.4)	62–79	70.5 (5.2)	60–84	.79
	MoCA score (/30)	29.1 (1.1)	27–30	28.8 (1.4)	26–30	.28
	Sex (male, female)	9, 26	N/A	17, 23	N/A	.13
	Education (Low, Medium, High)	4, 13, 18	N/A	4, 14, 22	N/A	.95
	Site (SW, CA, BE)	19, 8, 8	N/A	20, 11, 9	N/A	.89
	Frailty score (0, 1, 2)	35, 0, 0	N/A	40, 0, 0	N/A	N/A
	4-IADL (0, 1, 2, 3, 4)	0, 0, 0, 0, 35	N/A	0, 0, 0, 0, 40	N/A	N/A
	TUG (s)	8.65 (1.28)	5.35–11.05	8.06 (1.33)	5.40–12.20	.06
	HADS – Anxiety (/21)	2.3 (1.8)	0–7	1.8 (1.7)	0.7	.24
	HADS – Depression (/21)	2.1 (1.8)	0–7	2.4 (1.8)	0–6	.60

*4-IADL* 4-Instrumental Activities of Daily Living, *BE* Belgium, *CA* Canada, *HADS* Hospital Anxiety and Depression Scale, *MoCA* Montreal Cognitive Assessment, *SD* standard deviation, *SFL* StayFitLonger, *SW* = Switzerland, *TUG* = Timed Up&Go

### Total sample

**Fig. 2:**
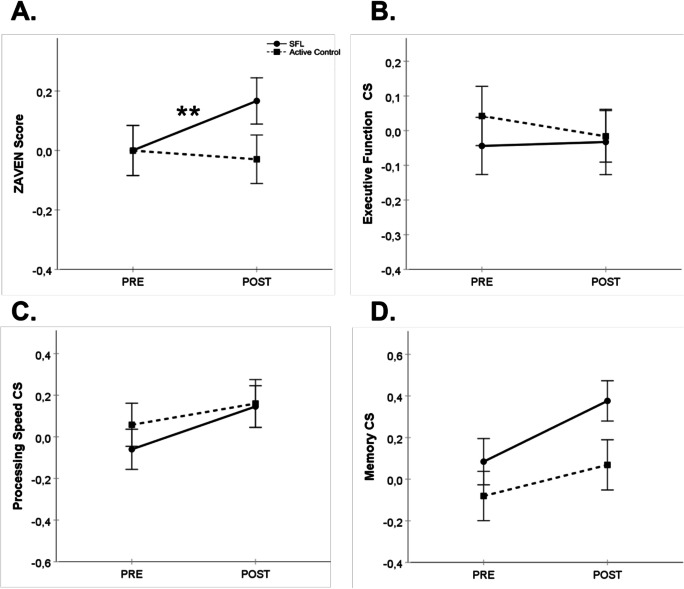
Performance on global cognition (**A**), executive function (**B**), processing speed (**C**), and memory (**D**) composite scores (mean ± SEM) at PRE and POST training for the SFL (full line, circles) and active control (dashed line, squares) interventions for the total sample of participants. Post hoc test results are reported here when a significant intervention × time interaction (*p* < .05) was observed. ** *p* < .01, mean POST–PRE change score for SFL intervention. CS, composite score; POST, post-training assessment; PRE, pre-training assessment; SEM, standard error to the mean; SFL, StayFitLonger

Figure 2 shows results for global cognition (Fig. 2A), executive function (Fig. 2B), processing speed (Fig. 2C), and memory (Fig. 2D) composite scores. For global cognition, the mixed model indicated no effect of intervention, *F*(1, 110.9) = 0.33, *p* = 0.57, or time, *F*(1, 109.7) = 2.20, *p* = 0.14, but there was an intervention × time interaction, *F*(1, 109.6) = 6.44, *p* = 0.01, effect size = 0.297. The estimated global cognition mean POST–PRE change score for the SFL intervention group was 0.14, 95% CI [0.04, 0.25]. The estimated global cognition mean POST–PRE change score for the active control group was − 0.04, 95% CI [− 0.14, 0.60].

The mixed model for executive function indicated no intervention, *F*(1, 109.8) = 1.47, *p* = 0.23, time, *F*(1, 111.1) = 0.37, *p* = 0.55, or intervention × time interaction, *F*(1, 110.9) = 0.12, *p* = 0.73. For processing speed, there was a time effect, *F*(1, 109.6) = 10.08, *p* = 0.002, but no intervention, *F*(1, 110.6) = 2.43, *p* = 0.12, or intervention × time interaction, *F*(1, 109.5) = 2.52, *p* = 0.12. The mixed model for the memory composite score indicated a time effect, *F*(1, 109.4) = 19.10, *p* < 0.001, but no intervention effect, *F*(1, 110.1) = 0.21, *p* = 0.65, or intervention × time interaction, *F*(1,109.2) = 1.98, *p* = 0.16.

### Pre-frail group

**Fig. 3:**
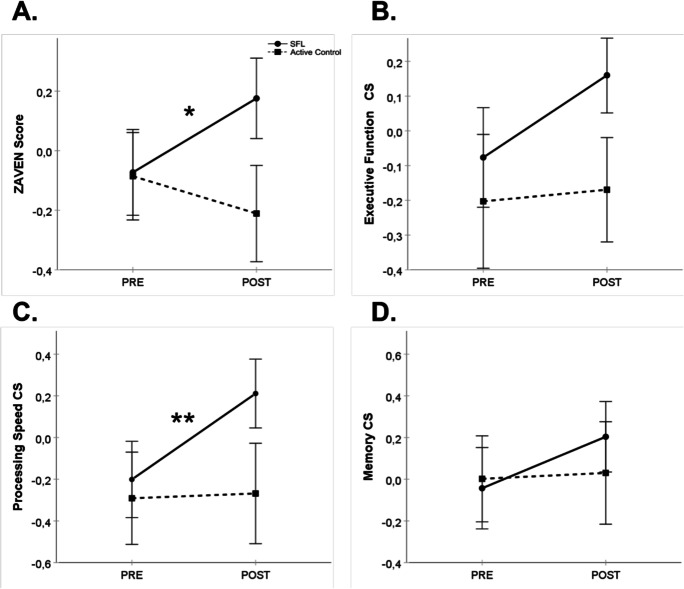
Performance on global cognition (**A**), executive function (**B**), processing speed (**C**), and memory (**D**) composite scores (mean ± SEM) at PRE and POST training for the SFL (full line, circles) and active control (dashed line, squares) interventions for the pre-frail participants. Post-hoc test results are reported here when a significant Intervention x Time interaction (*p* < .05) was observed. * *p* < .05 and ** *p* < .01, mean POST–PRE change score for SFL intervention. CS, composite score; POST, post-training assessment; PRE, pre-training assessment; SEM, standard error to the mean; SFL, StayFitLonger

Figure 3 shows the global cognition (Fig. 3A), executive (Fig. 3B), processing speed (Fig. 3C), and memory (Fig. 3D) composite scores for the pre-frail group. An intervention × time interaction was expected to support a larger effect due to the SFL intervention.

For global cognition, the mixed model indicated no effect of intervention, *F*(1, 35.6) = 0.01, *p* = 0.92, or time, *F*(1, 37.6) = 0.18, *p* = 0.68, but there was an intervention × time interaction, *F*(1, 37.5) = 7.18, *p* = 0.01, effect size = 0.451. The estimated global cognition mean POST–PRE change score for the SFL intervention group was 0.20, 95% CI [0.01, 0.38]. Estimated global cognition mean POST–PRE change scores for the active control group was − 0.14, 95% CI [− 0.32, − 0.04]. For the executive function composite score, there was no intervention effect, *F*(1, 35.9) = 0.06, *p* = 0.81, time effect, *F*(1, 38.3) = 0.99, *p* = 0.32, or intervention × time interaction, *F*(1, 38.2) = 0.48, *p* = 0.49. The mixed model for the processing speed composite score indicated no Intervention effect, *F*(1, 35.9) = 0.001, *p* = 0.97, but there was a time effect, *F*(1, 37.6) = 4.08, *p* = 0.05, and an intervention × time interaction, *F*(1, 37.5) = 7.41, *p* = 0.01, effect size = 0.379. The estimated processing speed mean POST–PRE change score for the SFL intervention group was 0.38, 95% CI [0.15, 0.62]. The estimated processing speed mean POST–PRE change scores for the active control group was − 0.06, 95% CI [-0.29, 0.17]. The mixed model for the memory composite score indicated no effect of intervention, *F*(1, 36.2) = 0.73, time, *F*(1, 38.0) = 1.82, or intervention × time interaction, *F*(1, 37.9) = 1.40.

### Robust group

**Fig. 4:**
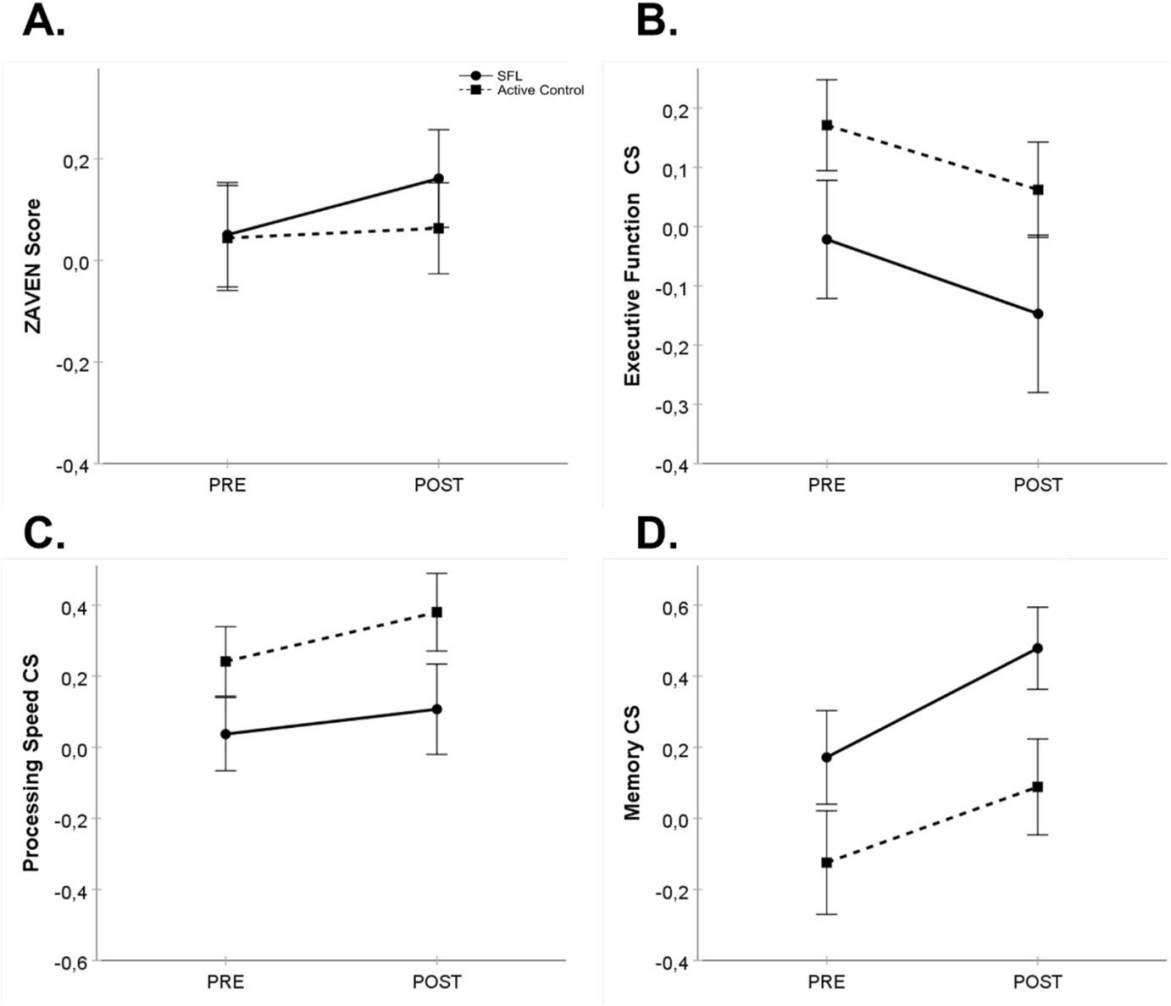
Performance on global cognition (**A**), executive function (**B**), processing speed (**C**), and memory (**D**) composite scores (mean ± SEM) at PRE and POST training for the SFL (full line, circles) and active control (dashed line, squares) interventions for the robust participants. CS, composite score; POST, post-training assessment; PRE, pre-training assessment; SEM, standard error to the mean; SFL, StayFitLonger

Figure 4 shows results for the global cognition (Fig. 4A), executive function (Fig. 4B), processing speed (Fig. 4C), and memory (Fig. 4D) composite scores. For global cognition, the mixed model indicated no effect of intervention, *F*(1, 66.2) = 0.52, *p* = 0.47, time, *F*(1, 70.4) = 2.18, *p* = 0.14, or intervention × time interaction, *F*(1, 70.4) = 1.18, *p* = 0.28. The estimated global cognition mean POST–PRE change score for the SFL intervention group was 0.11, 95% CI [− 0.02, 0.24]. The estimated global cognition mean POST–PRE change scores for the active control group was − 0.02, 95% CI [− 0.10, 0.13]. The mixed model for the executive function composite score indicated an intervention effect, *F*(1, 65.5) = 4.04, *p* = 0.048, but no time, *F*(1, 70.6) = 2.52, *p* = 0.12, or intervention × time interaction, *F*(1, 70.6) = 0.04, *p* = 0.85. The mixed model for the processing speed composite score indicated an intervention effect, *F*(1, 66.4) = 5.73, *p* = 0.019, and a time effect, *F*(1, 70.4) = 5.64, *p* = 0.02, but no intervention × time interaction, *F*(1, 70.4) = 0.83, *p* = 0.77. The mixed model for the memory composite score indicated a time effect, *F*(1, 69.8) = 18.84, *p* < 0.001, but no intervention, *F*(1, 65.8) = 1.37, *p* = 0.25, or intervention × time interaction, *F*(1, 69.8) = 0.74, *p* = 0.39.

The analyses on expectations showed no intervention × time interaction in the total sample, robust, or pre-frail groups, indicating that changes in expectations cannot account for the intervention effect (Supplementary Table 2). Regarding the controlled variables, we observed a site effect as participants from the Swiss site performed better than those in the Canadian and Belgian sites (Supplementary Table 3). We also found a sex effect in favor of women for the Zaven global cognition and memory scores.

## Discussion

This RCT assessed the efficacy of a computerized multidomain home-based intervention combining physical and cognitive exercises on the cognition of older adults. The ZAVEN global cognition score indicated a significant intervention × time interaction as the cognition of participants improved in the SFL intervention after training, unlike those in the active control condition. This finding is consistent with the FINGER study, which reported positive effects for a 2-year multidomain intervention on global cognition [3]. Unlike the FINGER study, which used usual care as a control, we used an active control condition where participants received physical activity guidelines and access to low-stimulation cognitive games. Furthermore, our study demonstrates a positive effect even though the duration is shorter than that of the FINGER study (6 months versus 2 years) and even though the intervention was provided remotely.

The positive effect found here deviates from the results summarized by Whitfield et al.’s [5] meta-analysis of four RCTs consisting of remotely delivered multidomain interventions, which reported no cognitive improvement. However, Whitfield et al.’s results should be interpreted with caution given the small number of studies. Furthermore, there are important differences between this study and those reviewed by Whitfield et al. One relates to the intervention content as the SFL intervention includes an individualized, progressive physical activity program with numerous illustrative videos, and empirically supported gamified cognitive exercises.

Another important aspect here was to examine effects on pre-frail individuals, who demonstrated a better response to training, which could have increased our ability to detect an intervention effect. Indeed, we found that pre-frail individuals randomized to the SFL intervention improved their global cognition and processing speed scores after the intervention, unlike participants randomized to the active control condition and unlike robust participants enrolled in either intervention. Thus, the effect observed when examining the entire group seems to be largely driven by the pre-frail participants, who showed a stronger response to this multidomain intervention. The effect found on the speed composite score might suggest that the improvement in processing speed drove the improvement in the ZAVEN global cognition score. Note, however, that there were benefits from the intervention when looking at the data from the other cognitive domains, even though these were non-significant. Hence, future research should focus on determining the cognitive domains that benefit most from similar interventions. The observed difference between pre-frail and robust individuals is consistent with the reserve/compensation hypothesis, which posits that vulnerable individuals are more likely to benefit from interventions designed to compensate for their difficulties, weaknesses or disabilities [13, 14]. There is some indication from prior studies that interventions may be beneficial to those who need them most, particularly if they are tailored to the characteristics of the target population (e.g., [40]). The physical exercises used here focused on strength and balance with a gradual, self-managed approach tailored to the sedentary older person. Similarly, our cognitive exercises were playful, which may be especially supportive for more vulnerable older adults. This underscores the importance of taking individual differences into account when designing and prescribing multidomain intervention programs.

The study has limitations that should be acknowledged: First, participants for the Belgian site were recruited and tested during the COVID-19 pandemic. Although we observed a site effect, this was due to performance of the Belgian and Canadian sites, being lower than the Swiss site. Thus, there is no indication for an effect specific to the Belgian site and no evidence that it modified the intervention effect. Second, frail individuals were excluded from our sample because we focused on prevention, but it could be interesting to examine whether the program has a positive effect on cognition in frail older adults. The sample size was estimated based on the physical outcome. Two of the composite scores used, executive and global cognition, showed low internal consistency. This indicates that they may reflect more than one cognitive construct. This was expected for the global cognition but not for the executive composite score. While it is an important issue, we did not include data on transfer to real-world daily functioning as our focus here was on cognition. Finally, the use of a purely computer-based intervention requires older adults to be technologically literate, which means that our group was biased toward those with technological skills.

In conclusion, we report positive effects of a multidomain remote intervention on cognition in older adults and propose that pre-frail older adults may benefit most from the program. One other important feature of the study was the use of a computerized program that allowed the intervention to be conducted entirely in the participantʼs home, which has rarely been done in past studies. Using a computerized remote approach has many benefits: It reaches a larger audience than face-to-face interventions, it is cost-effective in the long-term, it increases accessibility and flexibility, and it allows for personalization of the activities. The finding that more vulnerable older adults benefit most from an intervention to reduce cognitive decline provides support for public health interventions that encourage prevention strategies in older adults by specifically targeting a vulnerable population.

## Supplementary Information

Below is the link to the electronic supplementary material.

Supplementary file1 (DOCX 24 KB)

## Footnotes

1 Marker-by-treatment interaction http://www.bigted.org/NonAdaptiveDesigns/MarkerStratifiedDesigns.html
