# Correction to: Pre-frail older adults show improved cognition with *StayFitLonger* computerized home–based training: a randomized controlled trial

**DOI:** 10.1007/s11357-023-00868-5

**Published:** 2023-07-17

**Authors:** Sylvie Belleville, M. Cuesta, M. Bieler-Aeschlimann, K. Giacomino, A. Widmer, A. G. Mittaz Hager, D. Perez-Marcos, S. Cardin, B. Boller, N. Bier, M. Aubertin-Leheudre, L. Bherer, N. Berryman, S. Agrigoroaei, J. F. Demonet

**Affiliations:** 1 Research Centre, Institut Universitaire de Gériatrie de Montréal, CIUSSS du Centre-Sud-de-L’Île-de-Montréal, 4565, Queen-Mary Road, Montreal, Quebec H3W 1W5 Canada; 2 Université de Montréal, Montreal, Canada; 3 Leenaards Memory Centre and Infections Disease Service, University Hospital of Lausanne, Lausanne, Switzerland; 4 MindMaze SA, Lausanne, Switzerland; 5 HES-SO Valais-Wallis, School of Health Sciences, Loèche-les-Bains, Switzerland; 6 HES-SO Valais-Wallis, School of Management, Sierre, Switzerland; 7 Université du Québec à Trois-Rivières, Trois-Rivieres, Canada; 8 Université du Québec à Montréal, Montreal, Canada; 9 Montréal Heart Institute, Montreal, Canada; 10 Psychological Sciences Research Institute, Université catholique de Louvain, Louvain-la-Neuve, Belgium

**Correction to: GeroScience (2023) 45:811–822**

10.1007/s11357-022-00674-5

The original version of this article unfortunately contained an error in Table 1.

There is an error in the numbers (means and SD).

The corrected Table 1 is shown below.

**Table 1:** Participants’ demographic and clinical characteristics at baseline

Group	Characteristics	SFL		Active control		SFL vs Control
		Mean (SD) or N	Range	Mean (SD) or N	Range	*p* value
Total sample (SFL = 59 Control = 61)	Age (y)	70.6 (5.8)	61-82	72.0 (6.7)	60-94	.21
	MoCA score (/30)	29.1 (1.0)	25-30	28.8 (1.3)	26-30	.16
	Sex (male, female)	17, 42	N/A	24, 37	N/A	.22
	Education (Low, Medium, High)	6, 21, 32	N/A	6, 19, 36	N/A	.86
	Site (SW, CA, BE)	33, 15, 11	N/A	31, 17, 13	N/A	.85
	Frailty score (0, 1, 2)	35, 21, 3	N/A	40, 17, 4	N/A	.65
	4-IADL (0, 1, 2, 3, 4)	0, 0, 0, 0, 59	N/A	0, 0, 0, 0, 61	N/A	N/A
	TUG (s)	8.71 (1.23)	5.35-11.15	8.69 (1.95)	5.40-16.50	.95
	HADS – Anxiety (/21)	5.5 (2.7)	0-12	5.6 (3.1)	0-14	.35
	HADS – Depression (/21)	2.7 (2.3)	0-11	2.3 (2.6)	0-14	.57
Pre-frail (SFL = 24 Control = 21)	Age (y)	71.2 (5.5)	61-82	74.8 (8.2)	61-94	.09
	MoCA score (/30)	29.1 (1.0)	27-30	28.8 (1.2)	26-30	.39
	Sex (male, female)	8, 16	N/A	7, 14	N/A	1.00
	Education (Low, Medium, High)	2, 8, 14	N/A	2, 5, 14	N/A	.78
	Site (SW, CA, BE)	14, 7, 3	N/A	11, 6, 4	N/A	.83
	Frailty score (0, 1, 2)	0, 21, 3	N/A	0, 17, 4	N/A	.54
	4-IADL (0, 1, 2, 3, 4)	0, 0, 0, 0, 24	N/A	0, 0, 0, 0, 21	N/A	N/A
	TUG (s)	8.81 (1.18)	6.80-16.50	9.91 (2.36)	6.80-16.50	.05
	HADS – Anxiety (/21)	6.3 (3.0)	0-12	5.1 (3.1)	0-12	.91
	HADS – Depression (/21)	3.3 (2.8)	0-11	3.1 (3.6)	0-14	.30
Robust (SFL = 35 Control = 40)	Age (y)	70.2 (4.4)	62-79	70.5 (5.2)	60-84	.79
	MoCA score (/30)	29.1 (1.1)	27-30	28.8 (1.4)	26-30	.28
	Sex (male, female)	9, 26	N/A	17, 23	N/A	.13
	Education (Low, Medium, High)	4, 13, 18	N/A	4, 14, 22	N/A	.95
	Site (SW, CA, BE)	19, 8, 8	N/A	20, 11, 9	N/A	.89
	Frailty score (0, 1, 2)	35, 0, 0	N/A	40, 0, 0	N/A	N/A
	4-IADL (0, 1, 2, 3, 4)	0, 0, 0, 0, 35	N/A	0, 0, 0, 0, 40	N/A	N/A
	TUG (s)	8.65 (1.28)	5.35-11.05	8.06 (1.33)	5.40-12.20	.06
	HADS – Anxiety (/21)	4.9 (2.3)	0-9	5.9 (3.1)	0-13	.24
	HADS – Depression (/21)	2.3 (1.8)	0-7	1.8 (1.7)	0-7	.60

*4-IADL* 4-Instrumental Activities of Daily Living, *BE* Belgium, *CA* Canada, *HADS* Hospital Anxiety and Depression Scale, *MoCA* Montreal Cognitive Assessment, *SD* Standard Deviation, *SFL* StayFitLonger, *SW* Switzerland, *TUG* Timed Up&Go

