# Supplementary material for: Pre-frail older adults show improved cognition with StayFitLonger computerized home–based training: a randomized controlled trial
Source: GeroScience. 2022 Oct 21;45(2):811–22. doi: 10.1007/s11357-022-00674-5 (PMC9589849; doi:10.1007/s11357-022-00674-5)
Supplement: Supplementary file 1 — Supplementary file1 (DOCX 24 KB) [file 11357_2022_674_MOESM1_ESM.docx]

**Pre-frail older adults show improved cognition with *StayFitLonger* computerized home-based training: a randomized controlled trial**

S. Belleville^1,2*^, M. Cuesta, M. Bieler-Aeschlimann, K. Giacomino, A. Widmer, AG. Mittaz Hager, D. Perez-Marcos, S. Cardin, B. Boller, N. Bier, M. Aubertin-Leheudre, L. Bherer, N. Berryman, S. Agrigoroaei, JF. Demonet.

^1^Research Centre, Institut universitaire de gériatrie de Montréal, Montréal, Canada

^2^Université de Montréal, Montréal, Canada

**Corresponding author:**

Sylvie Belleville, PhD

^1^Research Centre, Institut universitaire de gériatrie de Montréal, Montréal, Canada

^2^Université de Montréal, Montréal, Canada

Email: [sylvie.belleville@umontreal.ca](mailto:sylvie.belleville@umontreal.ca)

**GeroScience**

**Supplementary Table 1.** Demographic and clinical characteristics at baseline for participants who withdrew from the study in comparison to those who completed the study

|  | **Completed the study (N = 101)** | | **Withdrew from the study (N = 19)** | | **Completed vs withdrew** |
| --- | --- | --- | --- | --- | --- |
|  | **Mean (SD) or N** | **Range** | **Mean (SD) or N** | **Range** | ***p* value** |
| Age (y) | 71.3 (5.8) | 60-94 | 71.7 (6.1) | 60-88 | .74 |
| MoCA score (/30) | 29.0 (1.2) | 26-30 | 29.0 (1.0) | 27-30 | .89 |
| Sex (male, female) | 38, 63 | N/A | 3, 16 | N/A | .06 |
| Education (Low, Medium, High) | 9, 33, 59 | N/A | 3, 7, 9 | N/A | .55 |
| Site (SW, CA, BE) | 54, 26, 21 | N/A | 10, 6, 3 | N/A | .82 |
| Frailty score (0, 1, 2) | 65, 29, 7 | N/A | 10, 9, 0 | N/A | .18 |
| 4-IADL (0, 1, 2, 3, 4) | 0, 0, 0, 0, 101 | N/A | 0, 0, 0, 0, 19 | N/A | N/A |
| TUG (s) | 8.73 (1.73) | 5.35-16.50 | 8.59 (1.02) | 7.58-10.62 | .75 |
| HADS – Anxiety (/21) | 2.4 (2.4) | 0-14 | 2.8 (2.7) | 0-9 | .47 |
| HADS – Depression (/21) | 2.9 (2.4) | 0-14 | 1.6 (1.1) | 0-3 | .11 |

4-IADL, 4-Instrumental Activities of Daily Living; BE, Belgium; CA, Canada; HADS, Hospital Anxiety and Depression Scale; MoCA, Montreal Cognitive Assessment; SD, Standard Deviation; SFL, StayFitLonger; SW, Switzerland; TUG; Timed Up&Go.

**Supplementary Table 2.** Participants’ expectations of the training

| Group | Intervention | Estimated mean (SD) PRE | Estimated mean (SD) POST | Intervention | Time | Intervention x Time |
| --- | --- | --- | --- | --- | --- | --- |
| Entire sample | SFL | 4.85 (1.36) | 4.56 (1.27) | *F*(1, 106.5) = 3.25  *p* = .07 | *F*(1, 101.8) = 16.4  ***p* < .001** | *F*(1, 102.6) = 1.53  *p* = .22 |
|  | Active Control | 4.67 (1.35) | 4.12 (1.28) |  |  |  |
| Pre-frail | SFL | 5.19 (1.57) | 5.03 (1.37) | *F*(1, 33.7) = 5.14  ***p* = .03** | *F*(1, 31.6) = 7.24  ***p* = .01** | *F*(1, 33.9) = 3.24  *p* = .08 |
|  | Active Control | 4.87 (1.52) | 4.10 (1.39) |  |  |  |
| Robust | SFL | 4.70 (1.32) | 4.32 (1.28) | *F*(1, 64.6) = 0.06  *p* = .80 | *F*(1, 66.5) = 9.87  ***p* = .003** | *F*(1, 66.5) = 0.09  *p* = .76 |
|  | Active Control | 4.68 (1.34) | 4.22 (1.29) |  |  |  |

**Supplementary Table 3.** Controlled variables for each cognitive outcome

| Outcome | Variable | Entire Sample | Pre-frail | Robust |
| --- | --- | --- | --- | --- |
| ZAVEN | Age | *F*(1, 111,5) = 19.68  ***p* < .001** | *F*(1, 35.9) = 6.23  ***p* = .02** | *F*(1, 65.6) = 8.11  ***p* = .01** |
|  | Sex | *F*(1, 108.7) = 10.75  ***p* < .001** | *F*(1, 35.1) = 1.12  *p* = .30 | *F*(1, 65.7) = 11.06  ***p* < .001** |
|  | Education | *F*(2, 111.2) = 1.06  *p* = .35 | *F*(2, 36.8) = 0.05  *p* = .95 | *F*(2, 65.8) = 1.78  *p* = .18 |
|  | MoCA | *F*(1, 110.7) = 15.03  ***p* < .001** | *F*(1, 36.9) = 5.18  ***p* = .03** | *F*(1, 66.0) = 9.28  ***p* < .001** |
|  | Site | *F*(2, 110.7) = 6.38  ***p* < .001** | *F*(2, 35.9) = 2.54  *p* = .09 | *F*(2, 65.6) = 8.11  *p* = .06 |
| Executive function | Age | *F*(1, 111,9) = 4.20  ***p* = .04** | *F*(1, 36.3) = 1.72  *p* = .20 | *F*(1, 64.5) = 0.33  *p* = .57 |
|  | Sex | *F*(1, 107.8) = 0.27  *p* = .61 | *F*(1, 35.3) = 0.27  *p* = .60 | *F*(1, 64.7) = 0.29  *p* = .59 |
|  | Education | *F*(2, 111.4) = 0.73  *p* = .49 | *F*(2, 37.5) = 0.04  *p* = .96 | *F*(2, 64.8) = 1.21  *p* = .30 |
|  | MoCA | *F*(1, 110.7) = 6.24  ***p* = .01** | *F*(1, 37.6) = 2.63  *p* = .11 | *F*(1, 65.1) = 3.35  *p* = .07 |
|  | Site | *F*(2, 110.6) = 2.82  *p* = .06 | *F*(2, 36.2) = 1.47  *p* = .24 | *F*(2, 65.6) = 1.81  *p* = .17 |
| Processing speed | Age | *F*(1, 111.6) = 23.46  ***p* < .001** | *F*(1, 36.1) = 7.16  ***p* = .01** | *F*(1, 65.8) = 9.47  ***p* = .003** |
|  | Sex | *F*(1, 109.5) = 2.52  *p* = .12 | *F*(1, 35.6) = 0.86  *p* = .36 | *F*(1, 65.9) = 3.16  *p* = .08 |
|  | Education | *F*(2, 111.5) = 0.0005  *p* = 1.0 | *F*(2, 36.7) = 0.30  *p* = .74 | *F*(2, 66.0) = 0.36  *p* = .70 |
|  | MoCA | *F*(1, 111.1) = 5.63  ***p* = .02** | *F*(1, 36.8) = 0.59  *p* = .45 | *F*(1, 66.2) = 6.53  ***p* = .01** |
|  | Site | *F*(2, 111.1) = 2.99  *p* = .05 | *F*(2, 36.1) = 2.32  *p* = .11 | *F*(2, 66.4) = 1.10  *p* = .34 |
| Memory | Age | *F*(1, 111.3) = 6.50  ***p* = .01** | *F*(1, 36.5) = 2.77  *p* = .10 | *F*(1, 65.2) = 4.54  ***p* = .04** |
|  | Sex | *F*(1, 108.9) = 11.13  ***p* = .001** | *F*(1, 35.8) = 0.62  *p* = .43 | *F*(1, 65.3) = 9.67  ***p* = .003** |
|  | Education | *F*(2, 111.0) = 0.21  *p* = .81 | *F*(2, 37.1) = 0.0005  *p* = 1.0 | *F*(2, 65.4) = 0.57  *p* = .57 |
|  | MoCA | *F*(1, 110.7) = 11.46  ***p* = .001** | *F*(1, 37.2) = 8.84  ***p* = .005** | *F*(1, 66.5) = 4.54  ***p* = .04** |
|  | Site | *F*(2, 110.6) = 23.87  ***p* < .001** | *F*(2, 36.5) = 2.77  *p* = .10 | *F*(2, 65.8) = 15.42  ***p* < .001** |

MoCA, Montreal Cognitive Assessment; ZAVEN, Z-score of Attention, Verbal fluency and Episodic memory for Nondemented older adults
